# Extreme signal amplitude events in neuromagnetic oscillations reveal brain aging processing across adulthood

**DOI:** 10.3389/fnagi.2025.1498400

**Published:** 2025-03-04

**Authors:** Vasily A. Vakorin, Hayyan Liaqat, Sam M. Doesburg, Sylvain Moreno

**Affiliations:** ^1^Department of Biomedical Physiology and Kinesiology, Simon Fraser University, Burnaby, BC, Canada; ^2^Royal Columbian Hospital, Fraser Health Authority, New Westminster, BC, Canada; ^3^School of Interactive Arts and Technology, Simon Fraser University, Surrey, BC, Canada

**Keywords:** ageing, magnetoencephalography, brain rhythms, temporal variability, skewed distributions, heavy tail distributions, extreme values, neuronal avalanches

## Abstract

**Introduction:**

Neurophysiological activity, as noninvasively captured by electro- and magnetoencephalography (EEG and MEG), demonstrates complex temporal fluctuations approximated by typical variations around the mean values and rare events with large amplitude. The statistical properties of these extreme and rare events in neurodynamics may reflect the limits or capacity of the brain as a complex system in information processing. However, the exact role of these extreme neurodynamic events in ageing, and their spectral and spatial patterns remain elusive. Our study hypothesized that ageing would be associated with frequency specific alterations in the brain’s tendency to synchronize large ensembles of neurons and to produce extreme events.

**Methods:**

To identify spatio-spectral patterns of these age-related changes in extreme neurodynamics, we examined resting-state MEG recordings from a large cohort of adults (*n* = 645), aged 18 to 89. We characterized extreme neurodynamics by computing sample skewness and kurtosis, and used Partial Least Squares to test for differences across age groups.

**Results:**

Our findings revealed that each canonical frequency, from theta to lower gamma, displayed unique spatial patterns of either age-related increases, decreases, or both in the brain’s tendency to produce extreme neuromagnetic events.

**Discussion:**

Our study introduces a novel neuroimaging framework for understanding ageing through the extreme and rare events of the neurophysiological activity, offering more sensitivity than typical comparative approaches.

## Introduction

Recordings of electromagnetic fields from the human brain are widely used to study neurophysiological processes and their characterize functional changes across typical and clinical mental states. Non-invasive techniques, such as electroencephalography (EEG) and magnetoencephalography (MEG), are commonly employed to capture these recordings. These methods predominantly measure the integrated activity of post-synaptic and transmembrane currents generated by thousands of neurons (Lopes da Silva, 2013). Historically, EEG and MEG recordings have been interpreted primarily as reflecting rhythmic brain activity, commonly referred to as neural oscillations (Buzsáki and Draguhn, 2004).

EEG and MEG studies have traditionally compared properties of neural oscillatory across experimental groups or conditions by analyzing metrics averaged over a fixed recording period. However, this approach may overlook key aspects of temporal variability, in particular, rare events with extreme values near the maximum. Empirical evidence indicates that many brain parameters, such as firing rates or synapse counts, follow skewed distributions (Buzsáki and Mizuseki, 2014). This pattern extends to both functional and anatomical features of the brain across multiple hierarchical levels (Buzsáki and Mizuseki, 2014; Roberts et al., 2015).

Skewed distributions suggest that single representative values, such as the mean or median, fail to capture the full range of temporal variability in brain activity (Limpert and Stahel, 2011). For instance, no single “average” neuron exists in a population because the minority of fast-firing neurons disproportionately influences the group’s mean firing rate. Similarly, neuronal ensemble sizes cannot be accurately described by an average value, as their distribution often follows, if not a pure power law, then at least a hybrid distribution combining power-law scaling and exponential decay (Dehghani et al., 2012). These findings underscore the need to account for the heavy-tailed nature of brain activity distributions when analyzing neural dynamics.

Multiple studies have demonstrated associations between temporal variability in neurophysiological signals and various cognitive, behavioral, and sensory processes (Basalyga and Salinas, 2006; McIntosh et al., 2008; Pinneo, 1966). Signal variability have been proposed as an indicator of effective and optimal performance (Faisal et al., 2008). However, despite the substantial body of research in this area, many studies continue to rely on traditional averaging approaches to analyze neurophysiological data (Garrett et al., 2013). These approaches present significant limitations as they fail to account for the inherently skewed distributions that characterize the variability of parameters governing the behavior of the underlying dynamical systems.

There is no consensus on which temporal properties of neural activity are most sensitive to variations in mental states (Cohen, 2017). Several theoretical frameworks have been proposed to capture different aspects of temporal dynamics. Key concepts in this literature include brain microstates (Musso et al., 2010), neuronal avalanches (Beggs and Plenz, 2003), and metastability (Tognoli and Kelso, 2014). Brain microstates are defined as brief periods of quasi-stable brain activity, thought to result from the coordinated activation of neural ensembles within specific networks (Vakorin et al., 2013). The theory of neuronal avalanches describes neurophysiological activity as cascades of bursts, supported by neural networks of varying sizes (Plenz and Thiagarajan, 2007). Finally, metastability characterizes a sequence of relatively stable states in a complex dynamical system. These states emerge from interactions among multiple parameters within the system’s phase space (Naik et al., 2017). Metastability offers a theoretical framework for understanding the balance between the brain’s stable activity patterns and its transient dynamics (Deco and Kringelbach, 2016).

These frameworks have been adapted to characterize age-related changes in temporal properties of neural oscillations in the typical brain across the lifespan (Kahana, 2006). Using the concept of microstates, Vakorin et al. (2013) explored age-related changes across early adolescence in resting-state EEG dynamics in eyes open and closed conditions under the framework of microstates. They showed that the number of microstates increased with age, whereas their average durations decreased. Similarly, Brookes et al. (2018) reported that when the number of microstates are held constant, the mean time spent in each state increased with age, spanning mid-childhood to early adulthood. Other studies have focused on the relationships between neurodynamic events and ageing. For rexample, Fosque et al. (2022) analyzed age-related changes in neural avalanche properties in adults across a wide age range.

Also, the concept of brain metastability has been used to quantify the signal complexity of neural oscillations. Signal complexity reflects the amount of information in neural signals, representing the collective activity of neuronal ensembles (Deco et al., 2017). Lippe et al. (2009) observed age-related increases in the complexity of resting-state EEG signals across infants and children. They also examined the relationship between maturation and signal complexity during a rapid face recognition task in children and young adults, reporting increased complexity with age.

In this study, we aimed to investigate extreme neural dynamics, characterized by the tails of skewed distributions of brain parameters, using skewness and kurtosis. Skewness quantifies the asymmetry of a probability distribution, while kurtosis measures the extremity of the tails without reflecting the shape of the central peak (Westfall, 2014). Both metrics capture properties of the signal tails, which may indicate periods of intense cognitive processing or heightened attention, as well as potential markers of cognitive impairment or neural dysfunction (Buzsáki and Mizuseki, 2014).

The tails of skewed distributions likely reflect moments of long-range synchronized neural activity, a process believed to play a critical role in both cognitive function and pathological conditions (Meijer et al., 2020; Nowak et al., 2017). Heightened synchronization during these moments may optimize neural processing but can also lead to pathological outcomes, such as seizures, when excessive (Jiruska et al., 2013). For example, several studies have examined extreme values in cortical oscillations under clinical conditions such as epilepsy, demonstrating their utility in facilitating seizure detection from EEG recordings (Xiang et al., 2020; Karpov et al., 2022). Other studies have identified differences in signal power distributions under varying conditions, such as the transitions between eyes-open and eyes-closed states, emphasizing the relevance of extreme values in characterizing the temporal variability of neurophysiological recordings (Mišić et al., 2011).

Statistical properties of distribution tails, such as skewness and kurtosis, provide practical insights into cognitive and clinical states. Skewness has proven effective in distinguishing cognitive states, such as meditation, mathematical problem-solving, and open-eye conditions, highlighting its potential for cognitive state monitoring (Joshua Davis et al., 2020). Similarly, kurtosis has demonstrated clinical relevance in multiple domains. In pediatric epilepsy, high-frequency brain signals exhibit significantly elevated kurtosis in patients compared to controls, particularly in epileptogenic zones (Xiang et al., 2020). This makes kurtosis a useful biomarker for identifying seizure onset regions and guiding clinical interventions. Also, kurtosis-based methods have been developed for detecting high-frequency oscillations in intracranial EEG, which are critical for localizing seizure onset zones in epilepsy patients (Quitadamo et al., 2018).

Kurtosis has also been implicated in neurodegenerative disorders. EEG studies in Alzheimer’s patients reveal higher kurtosis values compared to controls, leading to the development of kurtosis-based denoising techniques that enhance diagnostic accuracy (Liu et al., 2015). Furthermore, brain-computer interface (BCI) research has used kurtosis to classify motor imagery tasks in EEG data, achieving high accuracy in distinguishing between imagined left- and right-hand movements (Wu and Ye, 2006).

Empirical evidence on age-related changes in the maximal capacities of neurophysiological parameters remains limited, particularly when examined across the lifespan. In this study, we aimed to investigate brain ageing by focusing on extreme events in neurodynamics across adulthood, spanning young to elderly adults. We hypothesized that ageing would be associated with frequency-specific trajectories in the brain’s capacity to generate extreme neurodynamic events.

To quantify these age-related changes, we analyzed resting-state magnetoencephalography (MEG) recordings from the Cam-CAN repository (Shafto et al., 2014). Our analysis focused on frequency-specific temporal variations in MEG amplitude, assessing their statistical properties through the metrics of skewness and kurtosis. These metrics characterized the tails of the amplitude distributions, capturing extreme dynamics in brain activity. We then examined how these parameters evolved with age, identifying spatio-spectral patterns of age-related trajectories in extreme neurodynamics throughout adulthood.

## Methods

### Participants

We analyzed MEG data from the Cambridge Centre for Ageing and Neuroscience (Cam-CAN) Stage 2 cohort study (Shafto et al., 2014), which is a cross-sectional, multimodal, population-based adult lifespan (18–89 years old) investigation. The Cambridgeshire 2 Research Ethics Committee approved the Cam-CAN study which was conducted in compliance with the Helsinki Declaration. For secondary use of the Cam-CAN data, we obtained ethical approval from the Research Ethics Board (REB) at Simon Fraser University. We analyzed data from 646 healthy aging adults (see Shafto et al., 2014, for details on the inclusion and exclusion criteria). Participants were ranked according to their age and grouped into five age categories without an overlap, based on age percentiles, with 20% of participants in each group (19–36, 36–48, 48–61, 61–74, 74–89). The number of participants in the five age groups from young to elderly adults was 128 (69 females), 130 (60 females), 129 (65 females), 129 (60 females), and 130 (65 females).

### MEG data acquisition

The resting-state MEG data were collected as part of the CamCAN study (see Shafto et al., 2014; Taylor et al., 2017 for details on the study protocol and data acquisition). MEG was recorded with a 306-channel Elekta Neuromag MEG scanner (102 magnetometers and 204 planar gradiometers). During the MEG recording session, participants were asked to lie still and remain awake with their eyes closed for approximately 8–9 min. The recordings were sampled at 1 kHz, with a high-pass filter of 0.03 Hz. We analyzed minimally-preprocessed MEG from the Cam-CAN Release 005. These recordings were processed by the Cam-CAN team applying the MaxFilter 2.2.12 software (Elekta Neuromag Oy, Helsinki, Finland) without movement compensation. More specifically, a MaxFilter was applied to the continuous MEG data to remove noise from external sources (correlation threshold 0.98, 10-s sliding window) with temporal signal space separation (tSSS, Taulu and Simola, 2006), to remove mains-frequency noise (50-Hz notch filter), and to detect and reconstruct noisy channels.

### Neuromagnetic amplitude distributions

We analyzed the temporal variability in MEG signal fluctuations at five frequencies each assigned to one of five canonical frequency bands: 2 Hz (delta), 6 Hz (theta), 10.5 Hz (alpha), 22 Hz (beta), and 39 Hz (lower gamma). We analyzed data recorded by the MEG gradiometers only. We selected 11 non-overlapping segments for each participant, with each segment being 30 s long.

We processed each segment of the MEG time series by normalizing the signal to have a mean amplitude of zero and a standard deviation of one. We applied time-frequency decomposition to reconstruct frequency-specific MEG oscillations at five frequency points. Specifically, we used a complex Gaussian wavelet transformation of the eighth order, as described by Lee et al. (2019). This transformation allows for capturing a broader range of frequencies around each central frequency, enabling a more continuous scaling of the band-pass-filtered spectrum of neural activity within each canonical frequency band.

At each frequency and time point, we calculated the absolute value of the reconstructed analytic signal to evaluate fluctuations in the instantaneous amplitude of frequency-specific oscillations. To investigate the variability in signal amplitude across time, we derived empirical probability distributions that represent temporal variability in the neuromagnetic signal. Specifically, each 30-s time series was converted into an empirical distribution, where each time point corresponded to a single realization. From these distributions, we computed sample skewness and kurtosis.The skewness reflected a degree of asymmetry of distributions, whereas the kurtosis reflected a tendency to generate outliers (Westfall, 2014). Finally, for each participant, channel, and frequency, we averaged skewness and kurtosis values across segments by considering their median values. As a result of this procedure, each participant was associated with two arrays (one for skewness and one for kurtosis), representing the tailedness of the distributions of MEG signal across time: 204 MEG channels times five frequencies.

### Group analysis

For each of 11 MEG segments extracted from a channel, we calculated two characteristics, skewness and kurtosis (we used Fisher’s definition where a normal distribution has kurtosis = 0), of the corresponding power probability distribution. The median values of the characteristics across the eleven segments were used for further analysis.

**Figure 1:**
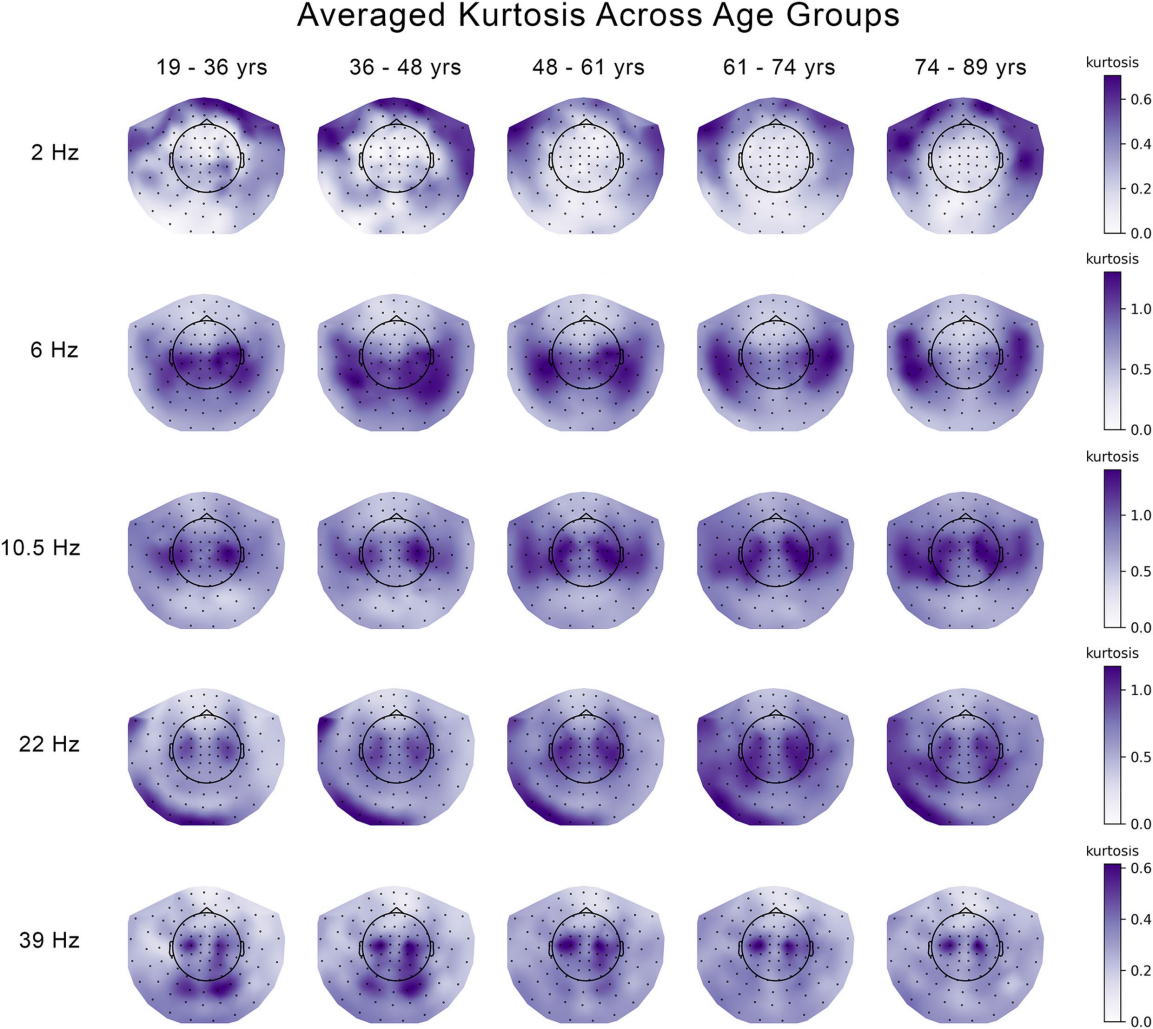
Changes in the kurtosis of the temporal variability of MEG signal across five age groups at 5 frequencies. The median kurtosis across eleven 30 s segments of temporal variability were averaged across subjects and plotted seperately for each age group and each frequency.

**Figure 2:**
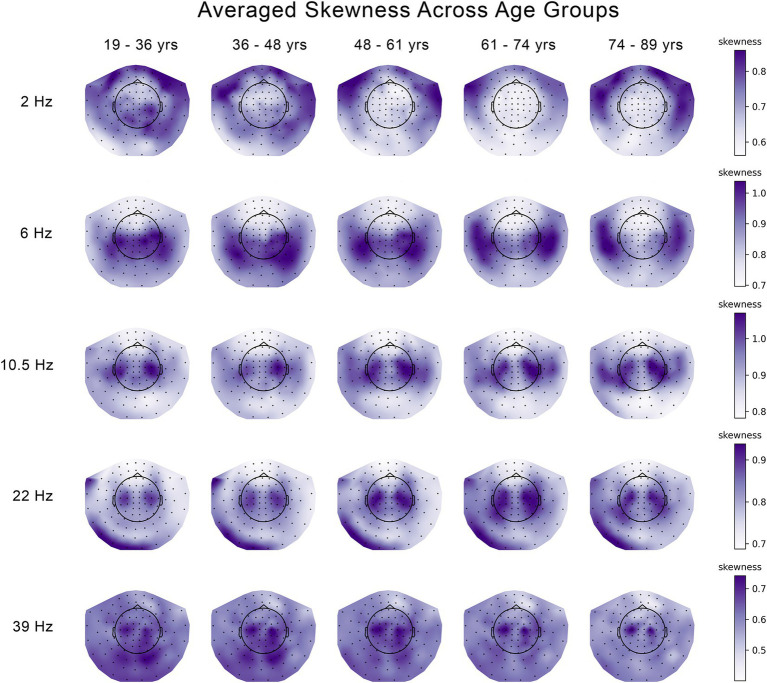
Changes in the skewness of the temporal variability of MEG signal across five age groups at 5 frequencies. The median skewness across eleven 30 s segments of temporal variability were averaged across subjects and plotted seperately for each age group and each frequency.

Differences across five age groups were explored separately for skewness and kurtosis, and separately for each frequency. Figures 1, 2 show the kurtosis and skewness values, respectively, across age groups, for each frequency. To test for differences in the skewness and kurtosis across age groups, we applied a multivariate analysis known in the neuroimaging and neurophysiology literature as Partial Least Squares (PLS). Specifically, we applied Mean-Centered PLS, wherein the overall group differences were tested without specifying *a priori* contrast (model) across the age groups (Krishnan et al., 2011; McIntosh and Lobaugh, 2004).

PLS considers all the data at once: all MEG features (the estimates of skewness or kurtosis for all MEG channels) and all five age groups. Mean-Centered PLS decomposes the covariance between the MEG data and dummy variables encoding five age groups with Singular Value Decomposition (SVD) into a set of latent variables (LV), similar to Principal Component Analysis. Each LV is associated with: (i) a vector of overall group contrast (a 5-dimensional vector in our case), (ii) a vector representing a contribution of each feature (MEG channel) to the identified group contrast, and (iii) a diagonal element of the middle matrix of SVD, which can used to quantify the variance explianed by a given LV. The significance of each group contrast is tested with a permutation test, based on the permutation of participants across the groups. This may be viewed as a global test, as the permutation test generates one *p*-value for one overall group contrast representing differences across the age groups across all features (MEG channels) at once. The robustness of the contribution of each feature is tested with a bootstrap test, based on resampling the participants with replacement within the groups.

We used 10′000 samples in the permutation and bootstrap tests. We considered the first LV, with the largest variance explained. As a result of this procedure, each PLS analysis was associated with: (i) an overall group contrast; (ii) the corresponding p-value; and (iii) a set of bootstrap ratio values (for each MEG channel), representing the robustness of contribution of each channel to the overall group contrast. The bootstrap ratio values are equivalent to z-scores. In our study, we used these terms interchangeably. For visualization purposes, z-scores were further averaged across MEG gradiometers from the same MEG sensor triplets. The spatial distributions of z-scores were visualized as topographic plots with the function plot_topomap from the MNE-python library (Gramfort et al., 2014). To visualize both negative and positive z-scores on the same plot, only for visualization purposes, we plotted the resulting z-scores as magnetometers, as the plot_topomap function applies the root mean square for a pair of gradiometers.

Note that large in magnitude z-scores reflect the most robust effect. In general, z-scores can be positive and negative. Positive z-scores directly support the overall group contrast. Negative z-scores can also support the contrast, but inversely. In case of negative z-scores, to interpret the directionality of the effects represented by the group contrast for the negative z-scores, we have to invert (multiply by −1) the contrast.

## Results

We performed 10 group analyses with PLS (five frequencies times two MEG measures), reporting the data-driven overall group contrasts with the largest variance explained. The group contrast was significant at all frequencies except for the kurtosis measure at lower gamma oscillations.

### Alpha rhythms

**Figure 3:**
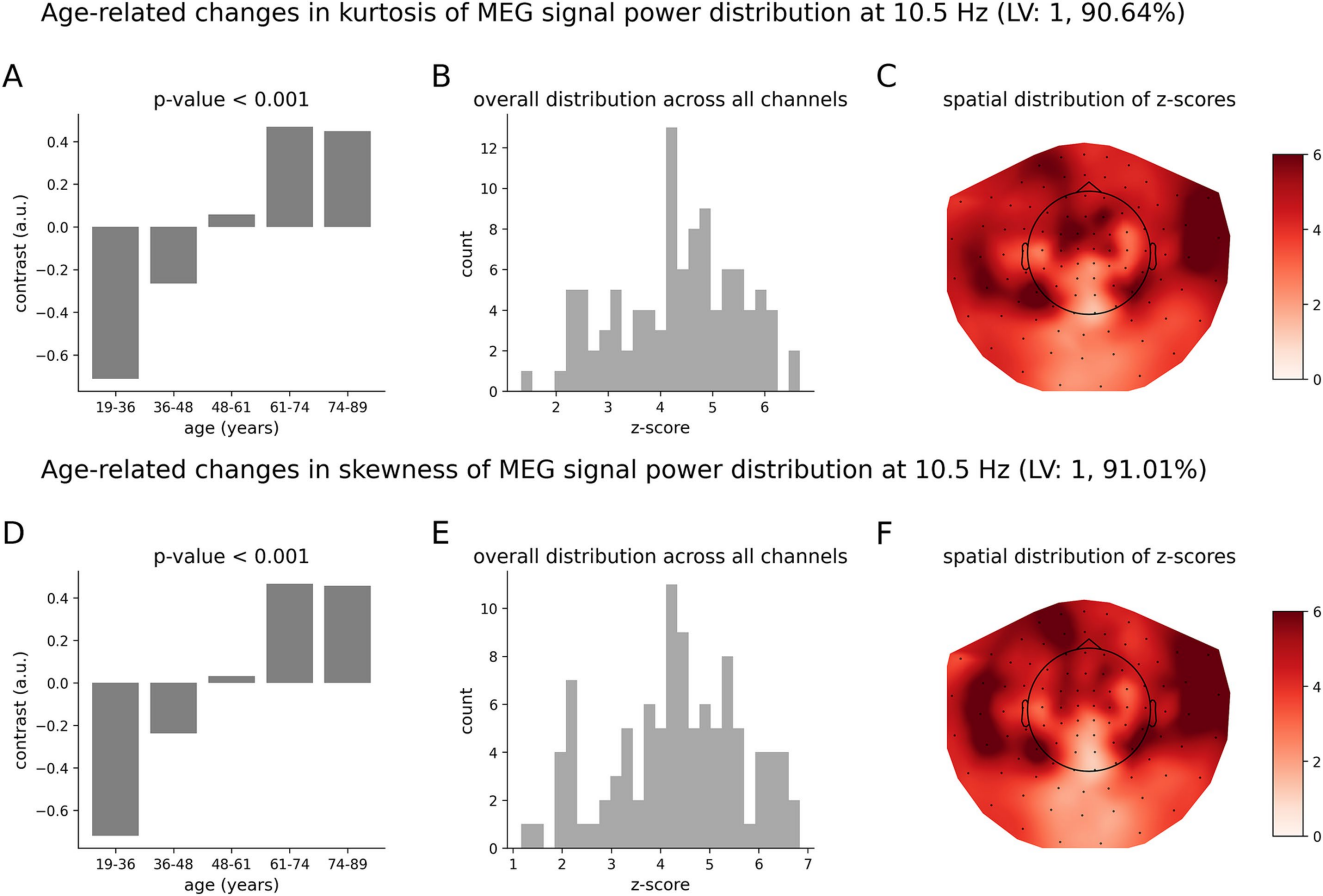
Age-related increases in the skewness and kurtosis of the temporal variability of MEG signal across five age groups at 10.5 Hz. **(A)** A data-driven overall contrast across the age groups for the skewness. **(B)** Corresponding distribution of z-scores, each associated with a MEG channel, showing the robustness of contribution of individual MEG channels to the overall contrast. **(C)** Same z-scores as in **(B)**, shown a spatial distribution of z-scores across MEG channels. **(D)** Overall contrast across five age groups for the skewness. **(E)** Corresponding distribution of z-scores for the skewness; and **(F)** same z-scores as in **(E)**, shown as a topographic map. The contrasts in panels **(A,D)** represent monotonic age-related changes in the skewness and kurtosis across the five age groups. The corresponding *p*-values reflecting the significance of overall group differences are provided in the titles of panels **(A,D)**. All the z-scores are positive, which implies that the skewness and kurtosis increase with age. The highest z-scores, which are shown in dark red, indicate the most robust effect.

Figure 3 shows the patterns of age-related changes in the kurtosis and skewness in temporal variability of alpha oscillations at 10.5 Hz. In general, these patterns are qualitatively and quantitatively similar. Specifically, Figures 3A,D illustrate the data-driven overall contrast across the five age groups for kurtosis and skewness, respectively. In both cases, the contrasts are found to be significant with *p* < 0.001. Both contrasts represent a trend of monotonic changes across the five age groups. The corresponding distributions of z-scores, each associated with one MEG channel, are shown in 31B and 3E, respectively. Note that these distributions are skewed toward positive values. This implies that, on average, the kurtosis and skewness of neuromagnetic signals’ temporal variability increase with age.

While the group contrast demonstrates the overall group differences, z-scores represent the robustness of the identified contrast across individual features (MEG channels). Figure 3C illustrates the same distribution of z-scores as shown in Figure 3B, only as a topographic map. Similarly, the distribution in Figure 3E is shown as a topographic map in Figure 3F. The largest positive z-scores, which are shown in dark red in Figures 3C,F, reflect the most robust effects across MEG channels. These effects are clustered around temporoparietal regions across both hemispheres.

### Delta rhythms

**Figure 4:**
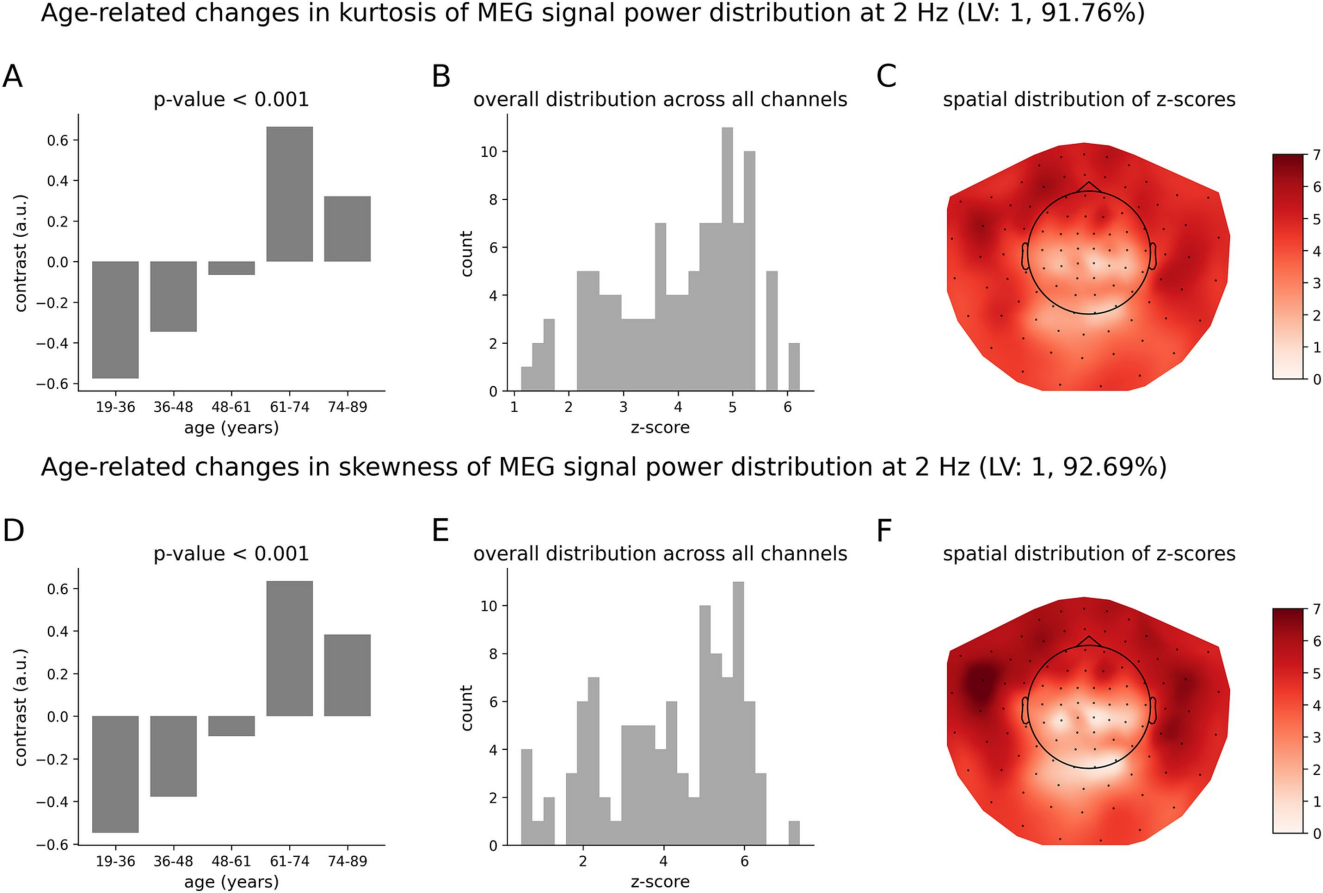
Age-related increases in the skewness and kurtosis of the temporal variability of MEG signal across five age groups at 2 Hz. **(A)** Overall group contrast for kurtosis; **(B)** corresponding distribution of z-scores; **(C)** same z-scores shown as the topographic maps; **(D)** overall contrast for skewness; **(E)** corresponding distribution of z-scores; (F) corresponding spatial distribution of z-scores. The contrasts in **(A,D)** represent age-related changes in skewness and kurtosis as an inverted U-function. All the z-scores are positive, which implies that the skewness and kurtosis increase across the age groups 19–36 to 61–74 years old (y.o.), and decrease for the age group 74–89 y.o. The highest z-scores indicate the most robust effect.

Similar to Figures 3, 4 shows the patterns of age-related changes in temporal variability of neurodynamics for delta oscillations at 2 Hz. Figures 4A,D illustrate the overall data-driven contrasts across the five age groups for kurtosis and skewness, respectively. In both cases, the contrast was found significant with *p* < 0.001. The corresponding distributions of z-scores, each associated with one MEG channel, are shown in Figures 4B,E, respectively. As z-scores are all positive, we interpret the group contrasts directly. Specifically, the group contrasts describe age-related changes as an inverted U-function. More specifically, the kurtosis (Figure 4A) or skewness (Figure 4D) of neurodynamics’s temporal variability first increases with age across the first four groups from 19 to 36 to 61–74 years old (y.o.), reaching a peak around 61–74 y.o., and subsequently going down for the elderly group (74–89 y.o). The topographic maps of positive z-scores in Figures 4C,F illustrate how the most robust effects are expressed across MEG channels. These effects are supported by clusters of MEG channels centered around frontal and temporal regions.

### Theta and beta rhythms

**Figure 5:**
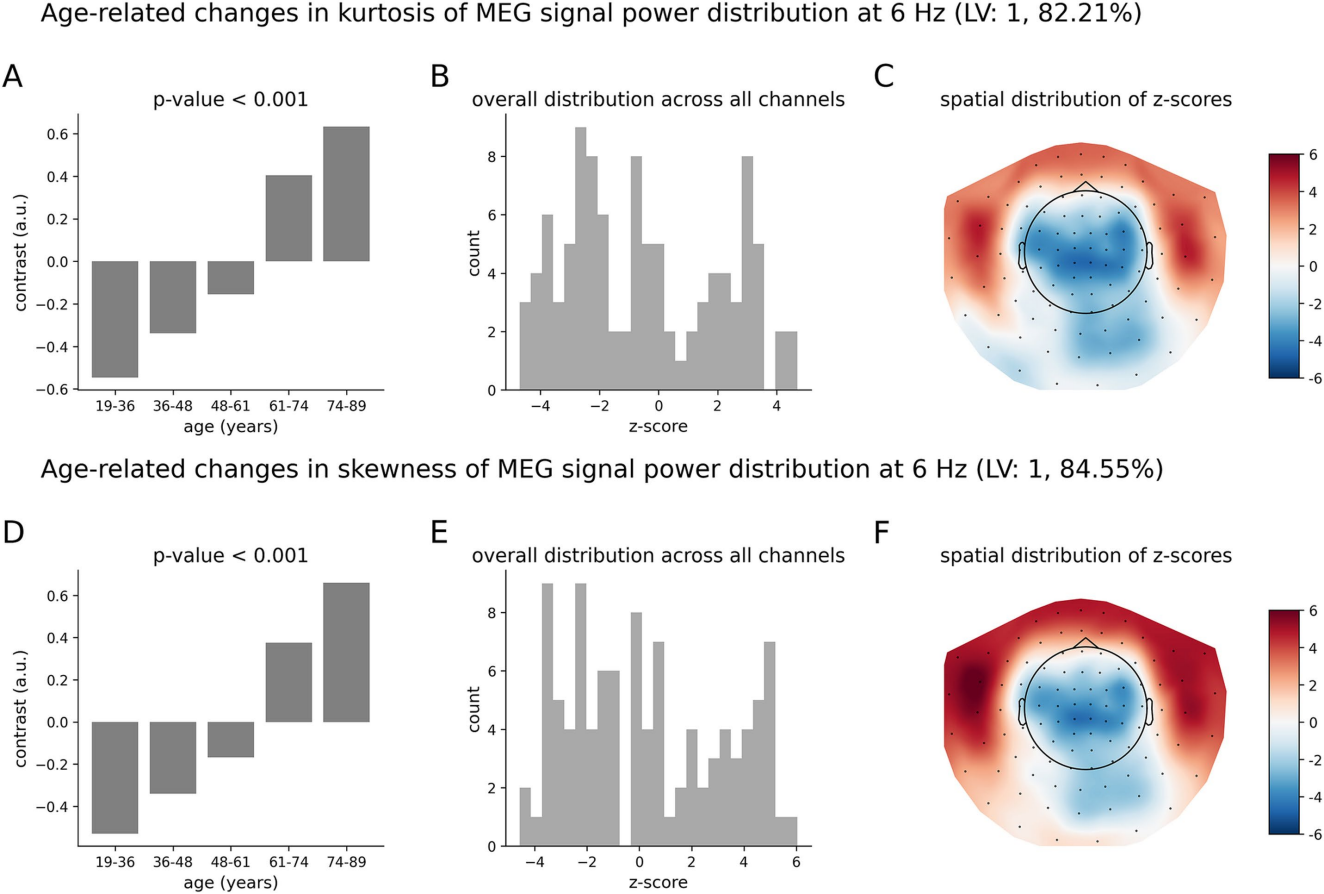
Age-related changes in the skewness and kurtosis of the temporal variability of MEG signal across five age groups at 6 Hz. **(A)** Overall group differences (group contrast) for kurtosis; **(B)** corresponding distribution of z-scores; **(C)** same distribution of z-scores shown as the topographic map; **(D)** overall group differences (group contrast) for skewness; **(E)** corresponding distribution of z-scores; **(F)** corresponding spatial distribution of z-scores. The contrasts represent a monotonic trend of age-related changes in skewness **(A)** and kurtosis **(D)**. The largest in magnitude z-scores, positive or negative, indicate the most robust effect. Positive z-scores indicate MEG channels, wherein the kurtosis and skewness and increase with age across the age groups according to **(A,D)**, respectively. Negative z-scores indicate MEG channels, wherein the skewness and kurtosis decrease with age across the age groups according to inversed group contrast (multiplied by −1) in **(A,D)**, respectively.

Similar to Figures 3–5 shows age-related patterns in the kurtosis and skewness of the distributions of MEG signal power across time for the theta oscillations at 6 Hz. The overall differences across age groups, as illustrated by the data-driven group contrast in Figure 5A for the kurtosis and in Figure 5D for the skewness, reflect a monotonic trend of changes with *p* < 0.001, similar to the monotonic trend found in the alpha frequency bend. At 6 Hz, however, the spatial distribution of z-scores is different from that at 10.5 Hz. Specifically, the distribution of z-scores at 6 Hz for both kurtosis (Figure 5B) and skewness (Figure 5E) include large in magnitude positive and negative z-scores. The increases in the kurtosis and skewness with age are mainly supported by the temporal regions, as shown by the positive z-scores in the topographic maps in Figures 5C,F, respectively. At the same time, decreases in the kurtosis and skewness due to aging are supported by the parietal regions, vertex, and occipital cortex, as can be seen by the negative z-scores in the topographic maps.

**Figure 6:**
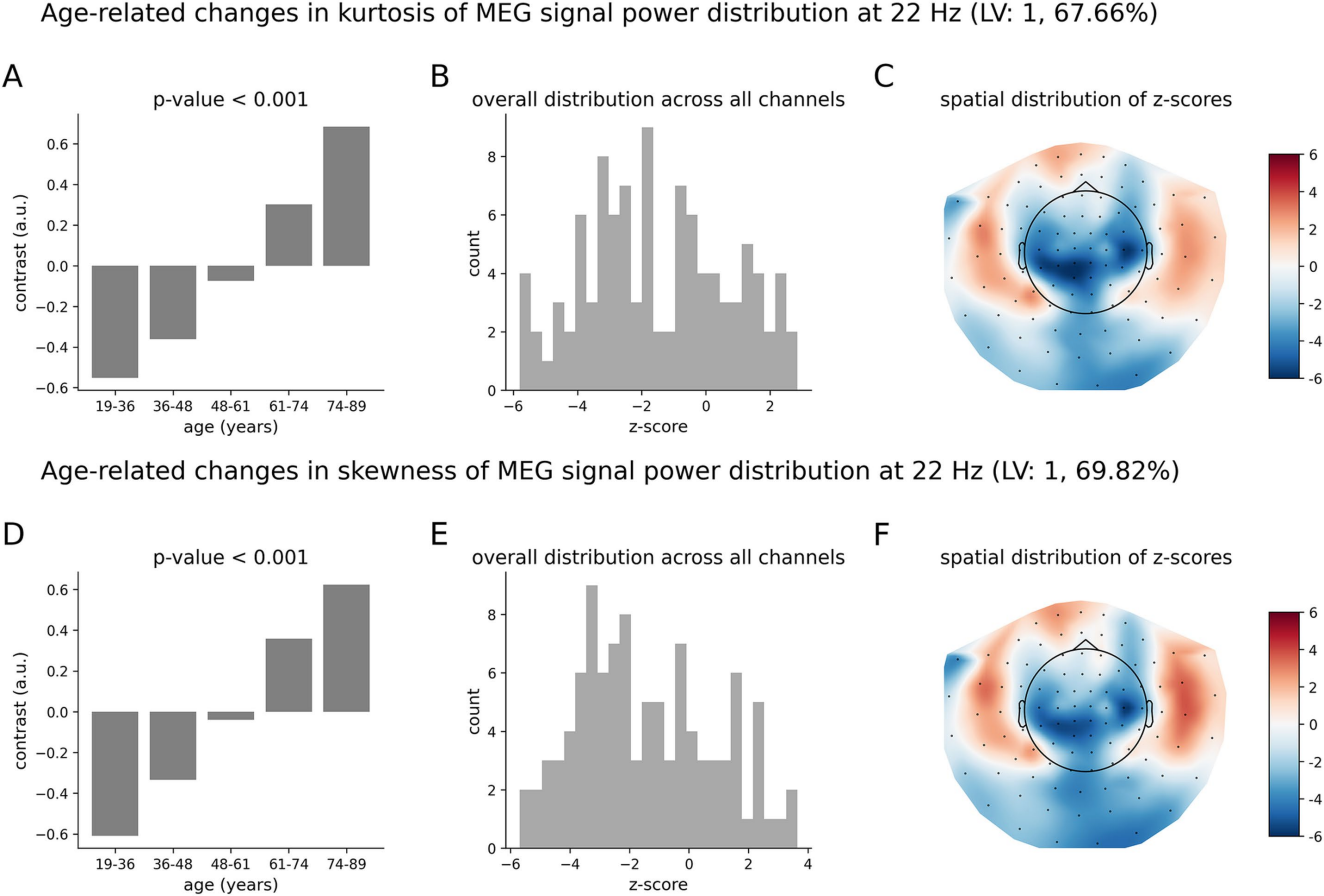
Age-related changes in the skewness and kurtosis of the temporal variability of MEG signal across five age groups at 22 Hz. **(A)** Data-driven overall group contrast for kurtosis; **(B)** corresponding distribution of z-scores; **(C)** corresponding spatial distribution of z-scores; **(D)** data-driven overall contrast for skewness; **(E)** corresponding distribution of z-scores; **(F)** corresponding spatial distribution of z-scores. The contrasts in **(A,D)** represent monotonic age-related changes of skewness and kurtosis. There is a mix of positive and negative z-scores, indicating brain areas of increasing and decreasing skewness and kurtosis in aging. Note that the spatial distributions of z-scores, as shown in **(C,F)** are visually similar to those shown in Figure 5.

The age-related pattern of increases and decreases in the tailedness of neuromagnetic variability at 6 Hz (Figure 5) is qualitatively and quantitatively similar to that for the beta oscillations at 22 Hz (Figure 6). The overall group contrasts significantly represent monotonic changes over the entire age range under consideration. The spatial distributions of z-scores (Figures 6C,F) at 22 Hz are similar to those at 6 Hz (Figure 5). Note, however, that the negative z-scores at 22 Hz by magnitude are larger than the positive z-scores: these distributions are skewed toward negative values. This implies that, on average, the effects represented by negative z-scores (decreases in the kurtosis and skewness) are more robust than those for positive z-scores (increases in the kurtosis and skewness). The corresponding distributions of z-scores at 6 Hz in Figures 5B,F are relatively symmetric, indicating that the robustness of contribution of brain regions expressing increases and decreases in the kurtosis and skewness are, on average, similar.

### Lower gamma rhythms

**Figure 7:**
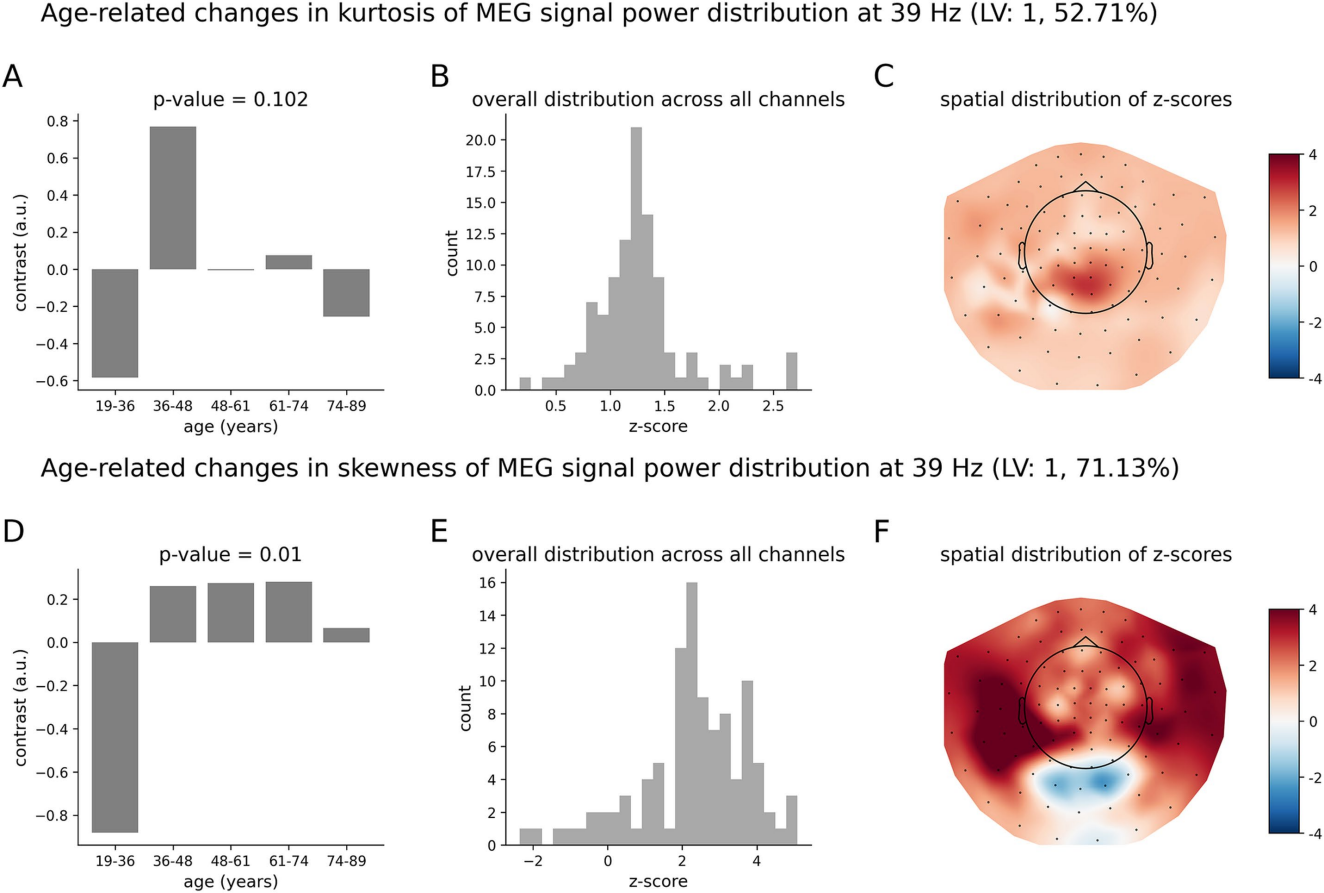
Age-related changes in the skewness and kurtosis of the temporal variability of MEG signal across five age groups at 39 Hz. **(A)** Overall group contrast for kurtosis; **(B)** corresponding distribution of z-scores; **(C)** same z-scores shown on the topographic plot; **(D)** data-driven overall group contrast for skewness; **(E)** corresponding distribution of z-scores; **(F)** corresponding spatial distribution of z-scores. The contrasts in **(A,D)** model age-related changes of skewness and kurtosis as an inverted U-shape. The overall group contrast for kurtosis was not significant. All the z-scores are positive, which implies that skewness increases across age and peaks at around 48–61 y.o and then decreases, whereas kurtosis peaks at 36–48 y.o and then decreases. The highest z-scores indicate the most robust effect.

Finally, Figure 7 shows the results for the lower gamma oscillations at 39 Hz. The overall group contrast (Figure 7D) representing differences in the skewness of neuromagnetic dynamics across age groups was found to be significant with *p* = 0.011. Such a pattern of age-related changes is supported by mostly positive z-scores (Figure 7E). This contrast models age-related changes as an inverted U-shape (Figure 7D). Specifically, the MEG measure of skewness first increases, reaching a peak around 61–74 years old, then decreasing with age. The group contrast for the kurtosis was not significant at the 95%-confidence interval, with a *p*-value of *p* = 0.102. We note that qualitatively, the contrast for the kurtosis (Figure 7A) also represents an inverted U-shape function, with a peak around 36–48 years old.

## Discussion

Our findings revealed that extreme events in the temporal variability of MEG signal amplitude, as quantified by skewness and kurtosis, exhibited distinct ageing trajectories across adulthood. These trajectories varied spectrally across the five canonical frequency bands: delta, theta, alpha, beta, and lower gamma. Furthermore, the spatial distributions of these age-related changes were region-specific, highlighting unique patterns of neurophysiological ageing across the brain. This study is the initial attempt to investigate the skewed nature of frequency-specific oscillations and to quantify empirical distributions of brain rhythm variability across the full adult lifespan, from young to elderly individuals. Importantly, we introduced a novel framework that focuses on extreme events in neurophysiological variability. This aspect is commonly overlooked in traditional neurophysiological studies, which typically emphasize average values and fail to capture the broader spectrum of temporal variability.

Our findings can be interpreted within the framework of the brain criticality hypothesis (O'Byrne and Jerbi, 2022). This hypothesis posits that optimal information processing in a healthy brain occurs at a phase transition point between synchronous and asynchronous, or regular and irregular, states. A brain operating near criticality can rapidly transition between these states, with small bursts of neurodynamic activity potentially giving rise to extreme value events. According to this framework, critical neural networks are essential for achieving optimal information transfer, which may explain the occurrence of extreme events in brain dynamics.

Our analysis revealed that the skewness and kurtosis of delta oscillations increased with age, indicating greater deviations from white noise in the form of extreme value events in MEG signal amplitude (Figures 4C,F). These findings align with recent work by Fosque et al. (2022), which investigated the brain’s functional organization across the lifespan under the framework of quasicriticality. Using the same MEG dataset from the Cam-CAN repository, Fosque et al. (2022) suggested that the brain operates near a line of maximal dynamic susceptibility, achieving a critical point for optimal information processing without external stimuli, noise, or dissipation. In this state, neuronal networks are activated successively through neuronal avalanches, whose size and duration follow scale-free or power-law distributions. Fosque et al. (2022) identified a positive correlation between the variance of avalanche size and age across adulthood. They did not consider individual frequency bands. However, considering that MEG spectral power approximately follows a 1/f distribution, the lowest frequencies, such as delta rhythms, are expected to significantly contribute to the reported correlations.

Further evidence supporting our results aronf the delta frequencies comes from Vakorin et al. (2013), who investigated age-related changes in adolescence by examining the non-stationarity of EEG rhythms as a sequence of quasi-stable patterns. They segmented EEG recordings into clusters of similar dynamics and quantified them in terms of the number of quasi-stationary microstates and their durations. They found that the mean duration of stationary EEG segments decreased with age, and these changes were correlated with variability in the signal power of delta oscillations. These findings suggest greater non-stationarity and complexity of delta oscillations during adolescence. Such a pattern resonates with our current results, which show an increase in extreme value events of delta oscillations with age. Collectively, these studies provide convergent evidence that changes in extreme value dynamics, particularly in lower frequency bands like delta rhythms, play a critical role in both developmental and ageing processes.

Our results revealed age-related changes in the theta (Figure 5) and beta rhythms (Figure 3), characterized by both increases and decreases in the skewness and kurtosis of temporal variability in neuromagnetic signal amplitude. The underlying spatial re-distribution of energy associated with these changes remains unclear. Previous research has linked similar spatial changes during ageing to a balance between localized neural processing and long-range communication (McIntosh et al., 2014). McIntosh and colleagues examined the signal complexity of EEG and MEG oscillations across young, middle-aged, and elderly adults. Using metrics such as entropy, they found that local entropy increased with age, which was associated with functional connectivity within each hemisphere. Conversely, distributed or global entropy decreased with age, particularly for interhemispheric connections.

Interestingly, in our study, the spatial patterns of age-related changes in the probability of extreme events for the theta and beta rhythms appeared visually similar. This observation may reflect cross-frequency coupling, a mechanism by which local dynamics could be integrated into long-range communication (Buzsáki and Watson, 2012). Cross-frequency coupling allows slower oscillations to propagate across broader spatial scales, facilitating neural coordination (Canolty and Knight, 2010). Specifically, coupling between theta and beta oscillations may play a critical role in sustaining healthy sensory networks (Cravo et al., 2011). This potential interaction between rhythms underscores the importance of exploring how cross-frequency dynamics contribute to changes in neural function across the lifespan. In addition to theta-beta coupling, due to the prominence of changes in alpha power across age (Broitman et al., 2024), we suggest that future studies also analyze coupling effects between alpha and other frequency bands, in the context of extreme events.

Our findings also can be considered in the context of previous studies investigating age-related changes in the aperiodic component of neural signals. This component is characterized by a 1/f spectral trend and is distinct from the rhythmic oscillations traditionally associated with EEG. The 1/f component is thought to reflect aperiodic neural activity that can influence the interpretation of EEG data, particularly in the context of brain network organization (Brake et al., 2024). Furtehrmore, it may reflect the baseline level of neural activity which has been linked to processing speed and cognitive decline in ageing populations (Tran et al., 2020), and more generally may be related to excitatory-inhibitory balance (Salvatore et al., 2024). The 1/f component has been shown to shift with ageing (Voytek et al., 2015). Since changes in the 1/f component likely influence the maximum values of neural signals, variations in the kurtosis and skewness of the signal may partially reflect this underlying shift in the aperiodic component. While our analysis focused explicitly on extreme events within the spectral bands, incorporating the 1/f exponent in future studies could provide a more comprehensive understanding of extreme neurodynamic events. This approach may help clarify how aperiodic shifts interact with rhythmic brain activity. Future research could benefit from using mathematical models that explain the generation of EEG signals (Brake et al., 2024).

We note that the two metrics we used in our analysis, skewness and kurtosis, produced similar results. These metrics are sensitive to extreme values, which makes them useful in neurological applications (Karpov et al., 2022; Xiang et al., 2020). For unimodal distributions such as the log-normal distribution, both skewness and kurtosis can be expected to reflect tail properties. Hence, it is not surprising that we found qualitatively similar results using both metrics in all frequency bands of interest, except for the gamma band. For instance, a previous study found that both higher skewness and kurtosis of MEG signals in three frequency bands (theta to lower gamma oscillations, ripples, and fast ripples) were associated with pediatric epilepsy compared to the control group (Xiang et al., 2020). Although it was not our explicit goal, our study confirms that skewness and kurtosis provide virtually identical results for our data set.

Our study has limitations. First, we used cross-sectional data to examine age-related changes, which limits our ability to quantify ageing trajectories. Cohort differences may influence the comparisons across age groups, as noted by Salthouse (2019). Second, our study does not account for the heterogeneity or sub-types within the ageing population, as recent research by Rodrigues et al. (2022), Nyberg et al. (2023), and Hinault et al. (2023) has highlighted. These studies suggest that the ageing population should not be treated as a homogeneous group, opening new avenues for future research. Potentially, our patterns of age-related changes may be affected by muscle artifacts. We aggregate our estimates by computing the median metrics across segments, which may help to mitigate the influence of artificial patterns within individual subjects.

In conclusion, our study introduces and validates a new framework focusing on extreme neurodynamic events to investigate brain processing across the adult lifespan. We found that ageing is characterized by shifts in the occurrence of large-amplitude neurodynamic events. These extreme events, measured through sample skewness and kurtosis of the MEG signal amplitude distributions, exhibit distinct temporal and spatial patterns across all frequency bands, from theta to lower gamma. These markers may offer a complementary view for defining age-related trajectories of healthy brain processing. By emphasizing extreme events rather than typical or mean values, our framework provides a novel approach to understanding brain function. Our findings highlight the potential of extreme events in neurodynamics for exploring maximal capacities, thereby contributing to a more comprehensive understanding of the neural mechanisms underlying ageing.

## Data Availability

Publicly available datasets were analyzed in this study. This data can be found here: Cam-CAN (Cambridge Centre for Ageing Neuroscience) dataset: https://camcan-archive.mrc-cbu.cam.ac.uk/dataaccess/.
